# Effect of long-term transcutaneous auricular vagus nerve stimulation in multiple system atrophy-cerebellar subtype: a case report

**DOI:** 10.3389/fnins.2024.1499793

**Published:** 2025-01-15

**Authors:** Zhao-Di Wang, Xiao-Ping Cheng, Zhen-Yi Liu, Di Wu, Jun Ni, Chuan-Juan Chen, Xin-Yuan Chen

**Affiliations:** ^1^Department of Rehabilitation Medicine, The First Affiliated Hospital of Fujian Medical University, Fuzhou, China; ^2^Department of Rehabilitation Medicine, The Affiliated Suzhou Hospital of Nanjing Medical University, Suzhou Municipal Hospital, Gusu School, Nanjing Medical University, Suzhou, China; ^3^Department of Radiology, The First Affiliated Hospital of Fujian Medical University, Fuzhou, China; ^4^Department of Neurology, The First Affiliated Hospital of Fujian Medical University, Fuzhou, China; ^5^Department of Nursing, The First Affiliated Hospital of Fujian Medical University, Fuzhou, China; ^6^National Regional Medical Center, Binhai Campus of the First Affiliated Hospital, Fujian Medical University, Fuzhou, China

**Keywords:** multiple system atrophy-cerebellar subtype, transcutaneous auricular vagus nerve stimulation, case report, ataxia, sleep

## Abstract

**Background:**

Multiple system atrophy-cerebellar subtype (MSA-C) is a predominance of cerebellar ataxia and autonomic failure. MSA-C has a rapid progression, with average 9 years from symptom onset to death. Despite its prevalence, there is still a lack of effective treatments. In recent years, it has been established that taVNS has significant therapeutic effects on epilepsy, depression, migraine, insomnia, and other diseases. Hence, we performed taVNS treatment for one MSA-C patient to explore whether taVNS could alleviate patient’s motor and non-motor symptoms.

**Case presentation:**

A 65-year-old woman diagnosed with MSA-C received taVNS treatment for the following duration and course: once a day, 40 min a time, 20 times a month continually for 12 months. Meanwhile, she received assessments of motor and non-motor symptoms at baseline, 4-weeks and 12-months after taVNS treatment. Motor symptoms assessments was made by Scale for the Assessment and Rating of Ataxia (SARA) and Unified Multiple System Atrophy Rating Scale (UMSARS), non-motor symptoms assessment by Pittsburgh Sleep Quality Index (PSQI), Hamilton Anxiety Scale (HAMA), and Hamilton Depression Scale (HAMD). After 4-weeks and 12-months of taVNS treatment, compared to baseline assessments, SARA scores decreased from 13 to 11 and then to 10.5, UMSARS scores from 28 to 24 and then to 23, PSQI scores from 19 to 13 and then to 6, HAMA scores from 13 to 3 and then remained unchanged, and HAMD scores from 7 to 4 and then remained unchanged.

**Conclusion:**

In the case, we found that short-term taVNS treatment can alleviate ataxia, sleep problem, anxiety and depression of the MSA-C patient. The effects can be maintained and some symptoms may be further improved after receiving long-term treatment. Our case report supports the feasibility and effectiveness of taVNS treatment in MSA-C patients.

## Introduction

Multiple system atrophy (MSA) is a disseminated and unexplained neurodegenerative disease that affects the extrapyramidal system, pyramidal system, cerebellum, and autonomic nervous system, with Parkinsonian symptoms, ataxia, and autonomic dysfunction as the main clinical manifestations (Foubert-Samier et al., 2020). According to prodromal motor symptoms, MSA is categorized into MSA parkinsonism variant (MSA-P) and MSA cerebellar variant (MSA-C) (Gilman et al., 2008). MSA-C with atrophy of medullo-ponto-cerebellar (MPC) white matter (WM) (Lu et al., 2014) is mainly characterized by gait ataxia, limb ataxia, cerebellar dysarthria, and cerebellar oculomotor disorders, which seriously affects the patient’s quality of life (Zhang et al., 2018). MSA-C has a rapid progression, with average 9 years from symptom onset to death, and there is still a lack of effective treatments (Terao et al., 2017). Therefore, how to improve patients’ motor and balance dysfunction through non-pharmacological treatment is the focus of current clinical research (Lamotte and Kaufmann, 2022).

It has been well established that transcutaneous auricular vagus nerve stimulation (taVNS) plays a vital role in neuromodulation, central projection, and anti-inflammatory pathway effects (Jiakai et al., 2023; Wang et al., 2021; De Smet et al., 2023), mainly by stimulating the branches of auricular vagus nerve (Peuker and Filler, 2002). The auricular branches of the vagus nerve project stimulation to the nucleus tractus solitarius (NTS). Then, the stimulation is projected extensively, directly or indirectly, to the reticular formation, forebrain, mesencephalon, limbic system, cerebellum, and other parts of the brain through other brainstem structures, such as the locus coeruleus (LC), parabrachial nucleus (PBN), and raphe nuclei (RN), in order to regulate the neural functional activities of these areas (Jiakai et al., 2023; Wang et al., 2021; De Smet et al., 2023; Butt et al., 2020). In recent years, taVNS has achieved significant therapeutic effects in epilepsy, depression, migraine, insomnia, and other diseases (Jiakai et al., 2023; Wang et al., 2021). Besides, treatment by means of taVNS is safe, non-invasive, easy to be applied clinically, and high in adherence (Kim et al., 2022; Tan et al., 2023).

Hence, we hypothesized that taVNS may provide beneficial assistance to MSA-C patients. However, there are no previous studies applying taVNS to patients with MSA-C. Therefore, we performed the taVNS treatment for MSA-C patient to explore whether taVNS could alleviate patient’s ataxia as well as other non-motor symptoms.

### Case presentation

**Figure 1:**
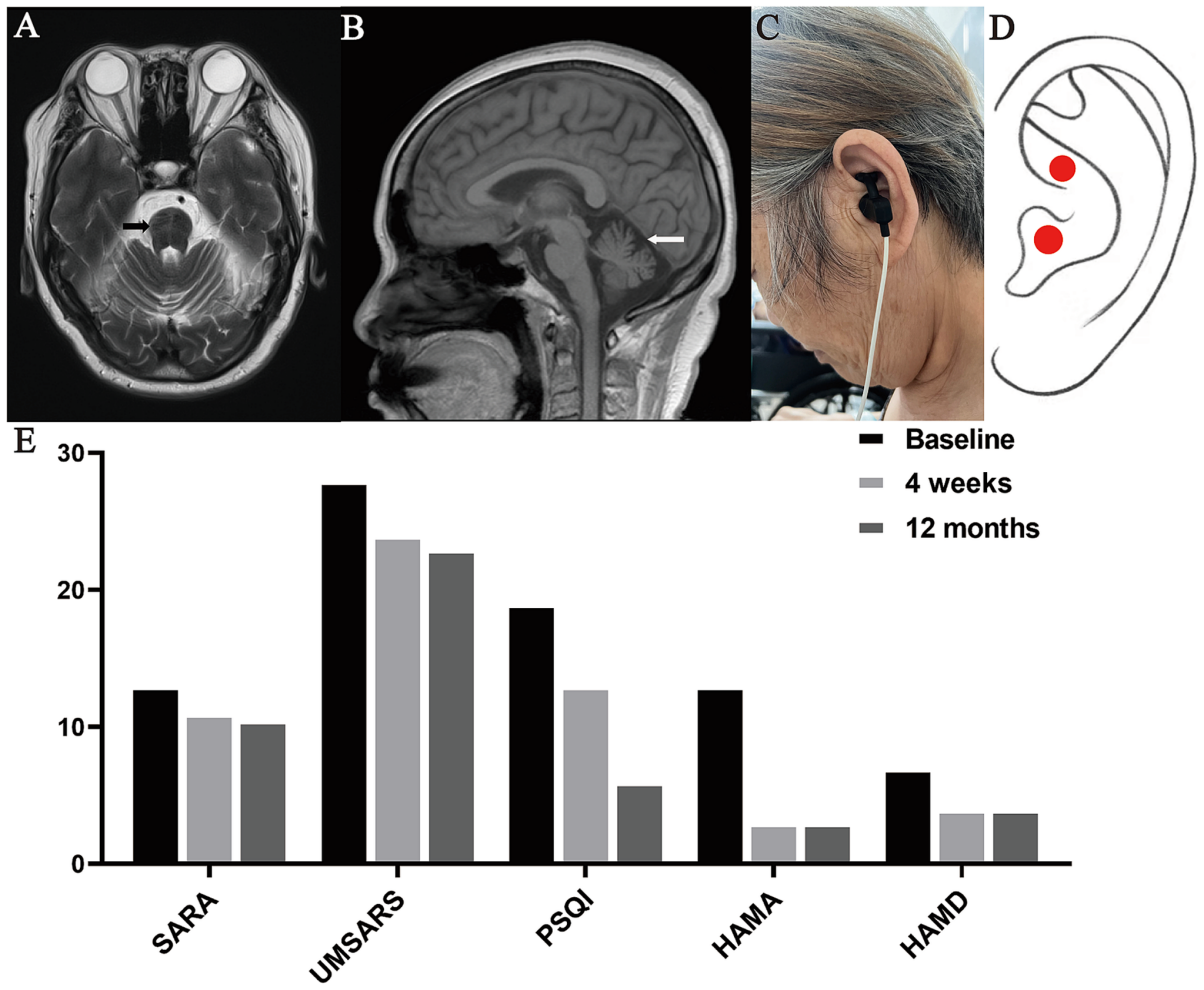
Representative images of the case. **(A)** T2W1 showed hot cross bun sign, **(B)** T2 showed cerebellar atrophy, **(C)** The patient with MSA-C was undergoing the taVNS treatment, **(D)** Position of the taVNS stimulation (cymba conchae), **(E)** Scores of SARA, UMSARS, PSQI, HAMA and HAMD at baseline, 4 weeks and 12 months after taVNS treatment.

A 65-year-old woman with MSA-C visited the Outpatient Rehabilitation Department of our hospital with walking instability for 1 year and worsening for 5 months. One year ago, the patient presented with unsteady walking without obvious triggers, accompanied by weakness of both lower limbs, which manifested as shuffling walking, difficulty in lifting the legs, and falling easily. Five months ago, the symptoms of unsteady walking and bilateral lower limb weakness became progressively worse. The patient also had slurred speech and frequent urination at night. Magnetic Resonance Imaging (MRI) of the cranium showed hot cross bun sign (Figure 1A) and cerebellar atrophy (Figure 1B). Nervous system examination of the patient showed that his consciousness is clear but the speech is slightly vague, and the remaining cranial nerve examination proved negative. The muscle strength of both upper limbs was grade 5, while that of both lower limbs was grade 4+. The muscle tone of the extremities was normal. The tendon reflex was symmetrical and active. Besides, no pathological signs were elicited. The left side of the body was unstable and inaccurate in the finger-nose test, while the right side being normal. The result of heel–knee-tibia test was also normal, with positive Romberg Sign and poor straight-line walking. In addition, proprioceptive sensation and superficial sensation were normal and Kernig’s sign is negative.

In addition, it was found that the patient had been suffering from hypertension for one year, and was taking Amlodipine on a regular basis, and her blood pressure was controlled at 110/80 mmHg. She had also been suffering from aortic sclerosis and cerebral ischemia for 5 months, and was prescribed oral Atorvastatin Calcium. At the same time, she took Buspirone Hydrochloride Tablets for improving ataxia.

In the case, the clinical and pharmacologic treatments the patient had previously received did not improve her symptoms and she experienced exacerbations. In order to alleviate the above symptoms, we tried to give the patient taVNS treatment, during which other former medication and lifestyle of the patient remained unchanged and the patient did not accept other therapies through the course. Informed consent has been signed with the patient. All research procedures were approved by Branch for Medical Research and Clinical Technology Application, Ethics Committee of the First Affiliated Hospital of Fujian Medical University (MRCTA, ECFAH of FMU [2022]400).

In this study, we used the taVNS device (Hwato, TENS-200A, Suzhou, China) to stimulate the left cymba conchae in patients with MSA-C (Figures 1C,D), which is in line with established practice (Farmer et al., 2020). The taVNS device used the following parameters: (i) Output pulse waveform: frequency 20 Hz (7 s) and 4 Hz (3 s), alternating between the two; (ii) Output pulse width: 0.2 ms; (iii) Output current limit ≤50 mA. The appropriate intensity was based on the patient experiencing a mild painful stimulation. During treatment, the patient was in supine or sitting position. The patient received taVNS in the Outpatient Rehabilitation Department for the following time and course of treatment: once a day, 40 min a time, 20 times a month for 12 months. Besides, she received assessments of motor and non-motor symptoms at baseline, 4-weeks and 12-months after taVNS treatment. Motor symptoms assessments included Scale for the Assessment and Rating of Ataxia (SARA) and Unified Multiple System Atrophy Rating Scale (UMSARS). Non-motor symptoms assessments included Pittsburgh Sleep Quality Index (PSQI), Hamilton Anxiety Scale (HAMA), and Hamilton Depression Scale (HAMD). Compared to baseline assessments, SARA scores decreased from 13 to 11 and then to 10.5, UMSARS scores from 28 to 24 and then to 23, PSQI scores from 19 to 13 and then to 6, HAMA scores from 13 to 3 and then remained unchanged, and HAMD scores from 7 to 4 and then remained unchanged (Figure 1E).

## Discussion

This case report firstly confirmed the efficacy of taVNS treatment in the patient with MSA-C. In the study, the patient suffered mainly from ataxia, walking instability for 1 year and worsening for 5 months. The pharmacologic treatments the patient had previously received did not improve her symptoms and some symptoms continued to progress. After receiving 12 months of taVNS treatment, she showed improvements in ataxia symptoms, sleep, anxiety and depression.

MSA-C is a neurodegenerative disorder with extrapyramidal system, pyramidal system, cerebellum, and autonomic nervous system (Lu et al., 2014; Terao et al., 2017), presenting with motor and non-motor symptoms such as ataxia, anxiety, depression and sleep disorders (Makawita et al., 2024). Cerebellar atrophy and decreased cerebellar blood flow are typical problems in MSA-C (Kimura et al., 2009). The cerebellum involves diverse functions from motor coordination to higher cognitive functions, impairment of which can cause ataxia and cerebellar cognitive affective syndrome (Lu et al., 2014). Previous studies have shown that the auricular branch of the vagus nerve, when stimulated by the taVNS, projects to the cerebellum through NTS (Jiakai et al., 2023; Wang et al., 2021; De Smet et al., 2023; Butt et al., 2020), and in turn causes significant changes of cerebral blood flow in posterior superior cerebellum (Chen et al., 2021). Also, taVNS can modulate the cerebello-thalamo-cortical pathway and the cerebellum may serve as an entry for modulating effects of taVNS (van Midden et al., 2023). Therefore, we infer that taVNS may improve ataxia symptoms of the MSA-C patient by modulating cerebellar function.

Sleep disorders are also one of the common symptoms in MSA-C patients, characterized by insomnia, rapid eye movement sleep behavior disorder (RBD), and daytime sleepiness (Lin et al., 2020). In the case, the patient had reduced total sleep time, decreased sleep efficiency, and prolonged time before sleep prior to the treatment, all of which improved after taVNS treatment. TaVNS may improve RBD of the patient by stimulating the nucleus of the solitary tract producing a long-lasting increase in the theta and beta band power (Martinez-Vargas et al., 2017). In addition, taVNS is a very promising resource for treating emotional disorders (Aranberri, 2024). It has also been applied to improve post-stroke depression and the depressive symptoms of COVID-19 with favorable results (Liu et al., 2024; Guo et al., 2021). Our study found that taVNS is effective in improving anxiety and depression in MSA-C patients, possibly by directly and indirectly modulating the activity and connectivity of key brain regions involved in anxiety and depression, inhibiting central and peripheral inflammation or modulating brain circuits via the hypothalamic–pituitary–adrenal axis (Aranberri, 2024; Guo et al., 2021).

In the above assessments, UMSARS is the most commonly used semiquantitative rating scale to assess symptoms and measure disease progression in MSA (Krismer et al., 2022). The higher the scale score, the more severe the condition (Krismer et al., 2022). MSA patients had a mean increase of approximately 4 points per year in UMSARS score with disease progression (Kaufmann et al., 2017). In the case, the UMSARS of MSA-C patient that received one-year taVNS treatment did not increase but instead decreased from 28 to 24 and then to 23. Also, no adverse effects were observed in the patients during treatment, and the safety of taVNS treatment was further validated. It can be seen that long-term adherence to taVNS treatment helped slow down disease progression and improve prognosis.

The above findings are based solely on this case. The assessment of the case lacked of objective parameters (such as gait analysis or the timed-up-and-go test) and objective markers (e.g., neuroimaging, electroencephalogram, biomarkers like inflammatory or oxidative stress markers), to provide more objective corroboration for our conclusions. Besides, the exact frequency, intensity, and duration of taVNS required for optimal therapeutic outcomes in MSA-C are not well-established. While the case report using a specific protocol (40 min a time, 20 times a month for 12 months) with reference to previous studies, it is unclear whether this protocol is the most effective. Further study is needed to standardize these parameters.

## Conclusion

In the case, we found that short-term taVNS treatment can improve ataxia, sleep, anxiety and depression of the MSA-C patient. The effects can be maintained and some symptoms may be further improved after long-term treatment. The findings in this case are of great interest for MSA-C patients with rapid disease progression and poor prognosis. However, taVNS parameters are diverse and it is not clear which one works best, so further validation is required. Our study is merely a case report, hence definitive conclusions cannot be drawn until further studies involving more evidence are done. In the future, there are still needs for myriad high-quality studies to demonstrate the group therapeutic efficacy and explicit treatment mechanisms of taVNS in patients with MSA-C to provide an effective, safe and promising treatment for them.

## Data Availability

The raw data supporting the conclusions of this article will be made available by the authors, without undue reservation.
